# Methods for Analyzing the Contents of Social Media for Health Care: Scoping Review

**DOI:** 10.2196/43349

**Published:** 2023-06-26

**Authors:** Jiaqi Fu, Chaixiu Li, Chunlan Zhou, Wenji Li, Jie Lai, Shisi Deng, Yujie Zhang, Zihan Guo, Yanni Wu

**Affiliations:** 1 Nanfang Hospital Southern Medical University Guangzhou China; 2 School of Nursing Southern Medical University Guangzhou China

**Keywords:** social media, health care, internet information, content analysis, big data mining, review method, scoping, online information, methodology

## Abstract

**Background:**

Given the rapid development of social media, effective extraction and analysis of the contents of social media for health care have attracted widespread attention from health care providers. As far as we know, most of the reviews focus on the application of social media, and there is a lack of reviews that integrate the methods for analyzing social media information for health care.

**Objective:**

This scoping review aims to answer the following 4 questions: (1) What types of research have been used to investigate social media for health care, (2) what methods have been used to analyze the existing health information on social media, (3) what indicators should be applied to collect and evaluate the characteristics of methods for analyzing the contents of social media for health care, and (4) what are the current problems and development directions of methods used to analyze the contents of social media for health care?

**Methods:**

A scoping review following Preferred Reporting Items for Systematic Reviews and Meta-Analyses guidelines was conducted. We searched PubMed, the Web of Science, EMBASE, the Cumulative Index to Nursing and Allied Health Literature, and the Cochrane Library for the period from 2010 to May 2023 for primary studies focusing on social media and health care. Two independent reviewers screened eligible studies against inclusion criteria. A narrative synthesis of the included studies was conducted.

**Results:**

Of 16,161 identified citations, 134 (0.8%) studies were included in this review. These included 67 (50.0%) qualitative designs, 43 (32.1%) quantitative designs, and 24 (17.9%) mixed methods designs. The applied research methods were classified based on the following aspects: (1) manual analysis methods (content analysis methodology, grounded theory, ethnography, classification analysis, thematic analysis, and scoring tables) and computer-aided analysis methods (latent Dirichlet allocation, support vector machine, probabilistic clustering, image analysis, topic modeling, sentiment analysis, and other natural language processing technologies), (2) categories of research contents, and (3) health care areas (health practice, health services, and health education).

**Conclusions:**

Based on an extensive literature review, we investigated the methods for analyzing the contents of social media for health care to determine the main applications, differences, trends, and existing problems. We also discussed the implications for the future. Traditional content analysis is still the mainstream method for analyzing social media content, and future research may be combined with big data research. With the progress of computers, mobile phones, smartwatches, and other smart devices, social media information sources will become more diversified. Future research can combine new sources, such as pictures, videos, and physiological signals, with online social networking to adapt to the development trend of the internet. More medical information talents need to be trained in the future to better solve the problem of network information analysis. Overall, this scoping review can be useful for a large audience that includes researchers entering the field.

## Introduction

### Background

Social media is an interactive service platform based on the internet that allows users to generate corresponding content, such as text posts or comments, digital photos or videos, and other interactively generated data, to establish connections with other users [1]. Therefore, social media can be regarded as an online promoter or enhancer of human networks [2]. At present, the number of social media users worldwide continues to grow at a faster rate than before the COVID-19 outbreak, with an increase of nearly 13.5 new users per second; driven by the double-digit annual growth rate, the number of social media users worldwide has reached 4.62 billion, and this current trend shows that in the next few months, this number will increase to 60% of the world's population [3]. Some popular social media websites have more than 100 million registered users, such as Facebook, TikTok, WeChat, Instagram, Weibo, Twitter, Tumblr, and LinkedIn [3].

Health care is the maintenance or improvement of health through the prevention, diagnosis, treatment, recovery, or cure of diseases, injuries, and other physical and mental disorders [4]. Social media has been widely used in health care fields. First, social media provides a new approach to disease prevention and management. Using social media to disseminate health information can achieve a wider range of influence than traditional public health publicity measures, while reducing economic and time costs [5,6]. Second, social media provides a platform for communication between patients and medical institutions. Through this platform, information and consultation can be provided to patient groups, and the impact of online platforms and patient satisfaction can also be evaluated in turn [7,8]. Third, social media provides a long-term interactive and continuously active platform between health care providers and patients, which could provide a way to achieve patient-centered continuous care of disease [9,10]. Patients can retrieve information through the network, share experiences, express themselves, seek support, learn skills, and vent their emotions so as to obtain peer emotion and social support and better adapt to the changes in the whole process of disease [11-13]. Finally, during the worldwide COVID-19 pandemic, through social media communication, the scientific community can cooperate globally in a faster way, understand the most important findings of the disease, and guide correct and useful information and knowledge to people seeking answers [14].

Social media has provided a new approach and mode in health care, showing great potential and playing an advantageous role, which deserves the attention of medical personnel. However, to the best of our knowledge, there is a lack of comprehensive reviews of methods for collecting and analyzing existing information on the internet. The main contributions of this review are as follows:

- Reviewing the focus and fields of existing relevant reviews to provide reference and comparison for selecting the research content of our own review
- Designing an extraction framework for methods to classify and describe them based on different properties from study methods, strategies, and different health care areas
- Analyzing and comparing the advantages and disadvantages of manual analysis and machine analysis
- Identifying the current issues and potential research directions and trends in the future

The rest of this scoping review is organized as follows. In the remaining part of the Introduction section, we describe previous related works and the motivation of this review. The search strategy, eligibility criteria, the process of study selection, and synthesis are explained in the Methods section. In the Results section, the review of our selected studies and their classification approaches are presented. The acquired outcomes, a detailed comparison, and limitations are provided in the Discussion section. Eventually, the main results, contributions, and implications of the review are discussed in the Conclusion section.

### Related Works and Motivation

Previous reviews on social media have mainly focused on its application for health care providers or patients [15-18], the relationship with mental health [19-22], the role in online health promotion [23-25], the features of social media health information [26-29], and big data analysis methods [30-33]. For example, Grajales et al [18] evaluated and synthesized 76 papers, 44 websites, and 11 reports, explaining and providing background knowledge on the application and potential realization of social media in medicine and health care according to 10 different social media categories. Karim et al [20] selected and evaluated the quality of 16 papers and analyzed the impact of social media activities on 2 mental health outcomes: anxiety and depression. In addition, Bazzaz et al [31] comprehensively reviewed the big data analysis methods in social networks and divided them into 2 categories: content- and network-oriented methods. To identify the role and influence of big data in social networks, the authors used the 5 Vs (volume, velocity, variety, veracity, and value) to describe the features of big data tools. Multimedia Appendix 1 shows the related works and details.

Only understanding the role and application of social media in the field of medical care cannot fully guide us to design a study to investigate the existing health management information on social media. Given the rapid development of social media and the increasing growth of social media users, effective extraction and analysis of the contents of social media for health care have attracted widespread attention from health care providers. Although a review by Bazzaz et al [31] synthesized the existing big data analysis methods for social networks, it did not focus on health care and only described the features of big data by using volume, velocity, variety, veracity, and value. Moreover, there was no mention of commonly used manual analysis methods in traditional qualitative fields, which makes it difficult to obtain a comprehensive understanding of the current methods for analyzing the contents of social media for health care. There is a lack of reviews to address the existing content on social media and research methods from the perspective of health care to make recommendations for future research and practice based on the current methodology. Therefore, this scoping review focuses on the health care content that users have accessed, shared, and generated and aims to collect and synthesize the methods for performing social media information research on health care. The research questions for this scoping review are:

- What types of research have been used to investigate social media for health care?
- What methods have been used to analyze the existing health information on social media?
- What indicators should be applied to collect and evaluate the characteristics of methods for analyzing the contents of social media for health care?
- What are the current problems and development directions of methods used to analyze the contents of social media for health care?

## Methods

### Search Strategy

PubMed, the Web of Science, EMBASE, the Cumulative Index to Nursing and Allied Health Literature (CINAHL), and the Cochrane Library were searched by 2 independent investigators (authors JF and WL) for the period from 2010 to May 2023. References and citations of the included literature were also traced. Our search strategy used the following terms: (1) “social media” OR “social network site*” OR Facebook OR twitter OR linkedin OR instagram OR weibo OR whatsapp OR telegram OR wechat OR “online community”, (2) “Delivery of Health Care” OR “health care” OR “nurs*” OR “health management”, and (3) “random*” OR “quantitative*” OR qualitative OR interview OR “clinical trial*” OR “surve*” OR “descriptive study” OR “cross-sectional stud*” OR “content analysis” OR phenomenology OR “grounded theory”. The search strategy was adapted in line with the query requirements of different databases (see Multimedia Appendix 2). Given the limited time and resources, we did not manually search conference proceedings, gray literature reports, and key journals.

### Eligibility Criteria

Studies related to the existing health management information on social media were initially included in this scoping review. Based on the inclusion and exclusion criteria (see Multimedia Appendix 3), we selected all types of original studies published in English, including experimental and observational methods. Studies published as abstracts of meetings or studies for which we were unable to obtain the full text after contacting the authors were excluded. The publication time of the studies was not limited.

### Study Selection and Synthesis

The study selection process was conducted following the Preferred Reporting Items for Systematic Reviews and Meta-Analyses (PRISMA) flow diagram. All retrieved citations were exported to Endnote for data deduplication and literature management. Three independent reviewers (authors JF, CL, and JL) screened the literature according to the eligibility criteria based on a standard study selection form. Any disagreements were resolved through discussion or by another reviewer (author YW).

After conducting a preliminary review of 10 randomly selected papers and discussing the applicability of the extraction scheme, we created a standard study selection form to extract the data of each study, including general information (author and publication date), characteristics of research objects (information type and sample size), and research content (research type, health care area, purpose, methods used for analyzing the contents of social media for health care, and results of the study). All data were extracted and verified by 4 reviewers (authors CL, SD, YZ, and ZG), and the extracted information was used for narrative synthesis. If we could not obtain data, we coded the variables in the form as “not reported.” Given the wide variety of study designs and the inconsistency of methods used in the studies, the extracted data could not be combined for meta-analysis. Hence, the extracted data were analyzed using narrative synthesis.

## Results

### Search Results

**Figure 1:**
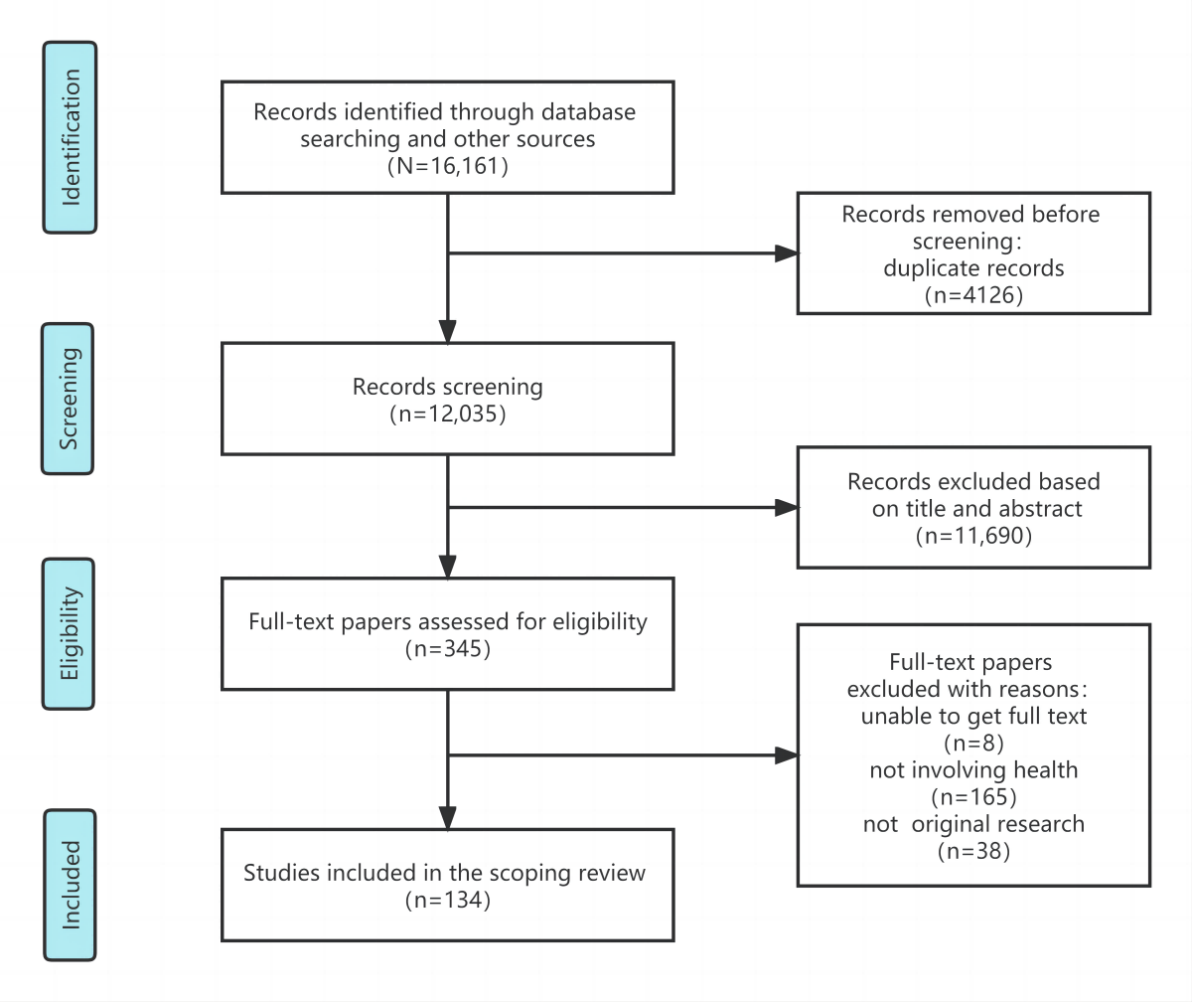
Literature screening process.

A total of 16,161 papers were identified after the database searching and reference and citation tracking. After checking for duplicates, screening titles and abstracts, and reading the full text, 134 (0.8%) papers were finally included [34-167]. The literature-screening process is shown in Figure 1.

### Description of the Studies

The 134 studies included were published between 2010 and 2023. A total of 90 papers (67.2%) were published within the past 5 years. There were 67 (50.0%) qualitative designs, 43 (32.1%) quantitative designs (including 40, 93.0%, cross-sectional studies; 2, 4.7%, longitudinal studies; and 1, 2.3%, cohort study), and 24 (17.9%) mixed methods designs.

**Figure 2:**
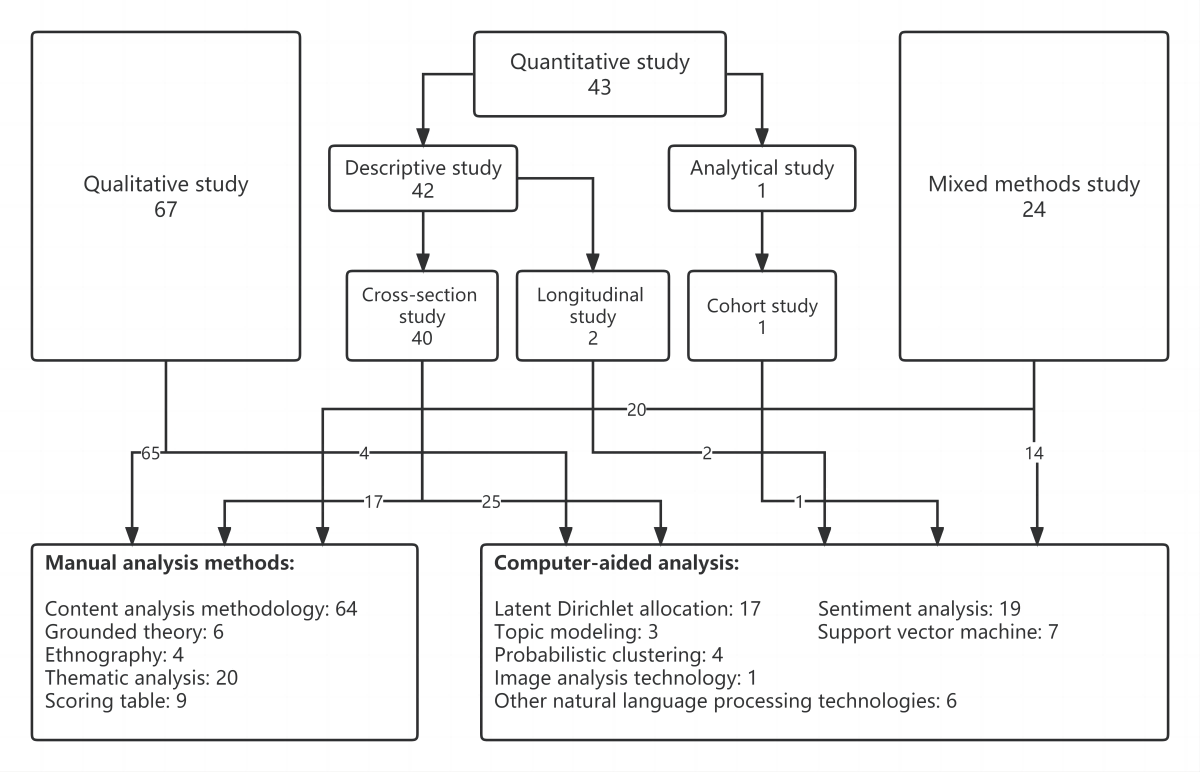
Features of included studies.

Of the 134 included studies, 102 (76.1%) used manual analysis methods to analyze health management information on social networking sites, including the content analysis methodology, grounded theory, ethnography, scoring tables, and thematic analysis, and 46 (34.3%) used computer-aided analysis tools to analyze the contents of social media, including latent Dirichlet allocation (LDA), support vector machines (SVMs), probabilistic clustering, image analysis, sentiment analysis, topic modeling, and other natural language processing (NLP) technologies. It should be noted that the research methods were not completely independent, and they could be used at the same time. Figure 2 shows the features of the included studies. Descriptions of these studies are presented in Multimedia Appendix 4.

### Aims of the Studies

The purpose of the included studies was divided based on the following 8 aspects: statistical demographic information (n=11, 8.2%); determining topics, including seeking support, information needs, and attitude (n=110, 82.1%); evaluating the role of social media (n=17, 12.7%); method improvement (n=11, 8.2%); providing medical or nursing insights (n=11, 8.2%); predicting trends (n=6, 4.5%); describing emotions (n=10, 7.5%); and looking for influencing factors (n=7, 5.2%).

### Manual Analysis Methods

#### Content Analysis Methodology

Of the 64 (47.8%) studies using the content analysis methodology, 35 (54.7%) [40,45,48,49,64,70,72,73,76,77,​^79^,^80^,^91^,^93^,^95^,^97^,^105^,^108^,^118^,^125^,^128^,^129^,^131^,^132^,^136^,​^138^,^149^,^150^,^153^-^155^,^159^,^161^,^162^,^166^] used general qualitative content analysis to classify and analyze online social media information. In logic application, 5 (7.8%) studies [78,83,100,109,152] used inductive content analysis to analyze the collected information and to ensure that the codes arose from the data themselves rather than being predefined. In addition, 3 (4.7%) studies [44,92,107] used deductive content analysis to apply coding strategies. Berkovic et al [57] applied summative content analysis through the encoding process, including counting and comparing content and then interpreting the underlying context. Scanfeld et al [133] applied Q-methodology to classify status updates. Furthermore, 2 (3.1%) studies [72,79] used the framework approach whose core was a series of interconnected stages. Thomas et al [95] put forward 5 established open-ended questions and then collected users' answers for descriptive content analysis to understand the supportive care needs of women with ovarian cancer at the end of treatment. Miller et al [154] combined the Health Belief Model to encode and analyze posts from patients with breast cancer. In addition, 2 (3.1%) studies [104,142] used quantitative content analysis to measure the frequency of content in papers in the entire sample and combined qualitative content analysis to facilitate the classification of information by source and content; 2 (3.1%) studies [86,144] combined machine coding and manual coding and compared their effectiveness; and in 1 (1.6%) study [59], the post content was preclassified and evaluated according to 8 aspects, such as emotion and media format, without mentioning the traditional content analysis steps.

#### Grounded Theory

Of 102 studies, 6 (5.9%) used grounded theory to analyze the contents of social media for health care. DeGroot [101] used grounded theory methods, including open coding, focused coding, and axial coding, to investigate the discourse on the wall of the memorial group and to identify the functions related to sadness in the discourse. In addition, 4 (66.7%) studies [35,129,157,158] built a topic framework through grounded theory and grouped similar data together to form topic categories; Haug et al [157] also explored patterns in data and accessed content using matrix-encoded queries to describe and compare provider attitudes toward naloxone treatment. Finally, Sun et al [89] used grounded theory to analyze 200 random samples to generate themes and compared the results with the overall analysis of LDA, which determined the outstanding concerns of patients with Crohn's disease.

#### Ethnography

Of 102 studies, 4 (3.9%) used ethnography to analyze the contents of social media for health care. Gajaria et al [155] analyzed the content of posts with the guidance of ethnology from high school or college students to understand young people's views on attention-deficit/hyperactivity disorder. Loeb et al [105] compared the posts of 2 communities over time using the linguistic ethnography method, revealing the obvious differences between the words used in 2020 and 2019, indicating an obvious behavioral change. Bridges et al [126] recruited 15 closed Australian breastfeeding association groups, captured all posts and comments, and divided them into different topic areas. Green et al [159] immersed themselves in 6 online health communities and, using the virtual ethnography method, developed a coding mode using an iterative deductive process to determine the subject category and further compared the similarities and differences between the 6 online health communities.

#### Thematic Analysis

Of the 20 studies using thematic analysis, 16 (80.0%) [38,49,50,53,65,78,90,91,113,122,127,134,140,145,150,163] used thematic analysis to analyze the content features of online information and extract topics. In addition, Oren et al [55] used grounded theory–based thematic analysis to examine qualitative data to determine the topic of tweets shared during the COVID-19 outbreak. Shah et al [51] sorted out the information published on the internet and conducted thematic analysis under the guidance of feminist disability theory to determine the aging effects and related themes in women at different ages. Hriberšek et al [103] conducted a thematic analysis of cleaned data after analyzing a large number of tweets using various computational methods and determined the parts of the patient's hospital journal. Furthermore, Çınar et al [52] conducted a modified thematic analysis of posts using open and axial coding methods.

#### Scoring Tables

Of 102 studies, 9 (8.8%) used scoring tables to analyze the contents of social media for health care. Kochan et al [67] evaluated searched groups and pages in terms of definitions, symptoms, risk factors, diagnosis, management, and prognosis according to the idiopathic pulmonary fibrosis–related content items supported by 30 prespecified guidelines, and assessed the quality of the posts through content scores. In addition, 2 (22.2%) studies [124,151] assessed the medical utility and relevance of content using DISCERN criteria and the Patient Education Materials and Assessment Tool (PEMAT) and evaluated misinformation using a Likert scale. Szmuda et al [34] also used DISCERN criteria to assess the quality and reliability of YouTube videos pertaining to narcolepsy. Temiz and Kandemir [141] used a 5-point Global Quality Scale (GQS) to evaluate the quality of videos and a 10-point scale to evaluate the content. The videos were classified as useful or useless based on their content and quality score. Furthermore, 2 (22.2%) studies [69,130] simultaneously used DISCERN criteria and the GQS to analyze YouTube videos of specific content, understanding the quality and benefits of the videos. Anastasio et al [94] used 2 separate scoring systems—DISCERN criteria and the Ankle Sprain Exercise Education Score (ASEES)—to evaluate the reliability, quality, and educational applicability of videos related to ankle sprain exercises. In addition, Ng et al [77] used the scoliosis-specific content score (SCSS) and DISCERN criteria to judge the quality of information on the internet pertaining to scoliosis.

### Computer-Aided Analysis

#### Latent Dirichlet Allocation

Of 46 studies, 17 (37.0%) used LDA to analyze the contents of social media, of which 10 (58.8%) studies [35,47,50,60,84,89,93,132,143,147] used LDA to identify the most common topics and classified and modeled clusters. In addition, 3 (17.6%) studies [46,68,91] compared the accuracy of LDA topic models using manual evaluation. For example, Jiang et al [68] cleaned up the text, adopted a dictionary related to medicine, added several specific terms particularly relevant to the analysis, and divided the words in the posts into different topics. In other aspects, Guntuku et al [63] used LDA topics as features to train a random forest classifier to distinguish language cues related to early pregnancy from those related to the first 3 months of pregnancy. Yang et al [98] modified LDA by adding a weighting scheme called conLDA and gathered virtual documents with a similar medical term distribution into conditional topics. Smith et al [45] tested several topic optimization methods to determine the optimal number of topics to be included in the model. Finally, Tangherlini et al [96] used LDA and contextual random walk traps (CRWT) to model posts as samples of hidden narrative frames to estimate the latent stories spreading on parenting social media sites.

#### Support Vector Machines

Of 46 studies, 7 (15.2%) used SVMs to analyze the contents of social media. Sofean and Smith [75] introduced machine learning classifiers (SVMs) to detect smoking behavior among Twitter users, found different smoking topics (eg, nicotine, smoking, and marijuana), and tested the accuracy of several different types of SVM models. Cui et al [146] compared the classification accuracy of influenza topics on Weibo between SVMs and the K-mean and verified the effectiveness of the method. Zhang et al [156] proposed a semisupervised learning framework to detect posts on drugs and their adverse reactions, extracted 7 independent features, identified the positive correlation between drug use and adverse reactions, and manually verified the accuracy. He et al [36] developed machine learning classifiers to identify and classify US tweets related to mask wearing during the COVID-19 pandemic so as to conduct subsequent qualitative content analysis. Kagashe et al [132] manually annotated random samples of tweets to generate features to be used by a machine learning classifier. The classifier was trained, evaluated, and applied to the entire data set to identify tweets indicating drug consumption. Next, a ranking list of widely used drugs and topics from those tweets was obtained. In Lao et al’s [165] research, uniformly trained researchers manually annotated the data set and extracted text features. These features were used in the SVM model and evaluated for performance through the area under the curve (AUC), precision, and recall. Myneni et al [135] used the distributed semantic method to generate the vector representation of all messages as input to a machine learning classifier, annotated the entire data set, and obtained the association measure between the given message and the previously determined communication topic so as to estimate the distribution of different types of online community content.

#### Probabilistic Clustering

Of 46 studies, 4 (8.7%) used probabilistic clustering to analyze the contents of social media. Yoon et al [164] applied the Newman clustering algorithm to group related tweets in the corpus. Manchaiah et al [80] divided the text content into smaller units, used the Reinert method to cluster the text fragments, then used a dendrogram characterizing the clustering to display the results, and finally carried out time series analysis. Tseng et al [148] preprocessed the text, used K-means clustering to cluster these converted problems into 10 separate clusters, calculated the similarity between them, and then manually explained and summarized the text of the top 3 problems in each cluster to obtain the most representative problems in each cluster. Lu et al [54] used the expectation maximization algorithm in various clustering technologies for stakeholder and topic identification and used the Rand index, the Jaccard similarity coefficient, and the Fowlkes-Mallows (FM) index to evaluate the clustering results.

#### Image Analysis

Only 1 (2.2%) study used image analysis technology. Zhang et al [81] preprocessed an image and automatically extracted several different types of information, including post coloring, post volume, frequency, and release time. Image content, characterization of pictures of people, and posted memes were then recorded to determine image features associated with postpartum depression.

#### Sentiment Analysis

Of 46 studies, 19 (41.3%) used sentiment analysis to analyze the contents of social media, of which 5 (26.3%) studies [43,60,82,161,164] analyzed the sentiment of posts using a value perception dictionary and an environmental reasoner (VADER). The predicted sentiment (composite score) was calculated by adding the valence scores of each word in the vocabulary, adjusting according to emotion-related rules, and then normalizing to obtain a value between –1 (the most extreme negative emotion) and +1 (the most extreme positive emotion). In addition, 1 (20.0%) of the 5 studies [161] also used TextBlob for thematic sentiment analysis. Xavier and Lambert [74] applies 3 lexicons (AFINN, Bing, and NRC), which were built around unigrams or single words, to comprehensively understand the positive or negative sentiment and trend in tweets. Crannell et al [88] used the Language Assessment by Mechanical Turk (LabMT) word list to perform a quantitative statistical analysis of a Twitter set of patients with a cancer diagnosis to investigate the relative sentiment (happiness) of the patients’ tweets. The frequency distribution of sentiment words was shown as a word change map. Berkovic et al [57] used Glaser and Strauss's 6 codes for sentimental analysis as a framework for emotional analysis of encoded tweets and applied emoji sentiment ranking to obtain information regarding the emotional content of the tweets. Sormunen et al [66] used the Gavagai tool, a lexical approach based on a list of sentiment-carrying terms, to quantify the basic emotions of each topic as positive or negative. Milley et al [104] used RStudio and the *sentiment* package to determine the overall sentiment of tweets. In addition, 2 (2.1%) studies [54,160] used sentiwordnet vocabulary to extract emotional terms from information and calculate their emotional scores. Cameron et al [85] used sentimental identification to identify expressions that contain emotions, including formal and slang words or phrases, and to assess the topic-related polarity of each emotional cue associated with a specific entity. Yang et al [98] modified the sentimental dictionary, extracted sentiment words, and accumulated the number of positive and negative sentiment words so as to analyze the sentiment tendencies of users using health communities (known as forums). Smith et al [45] used the *syuzhet* package to extract emotions and assigned each word to 8 emotions (eg, anger, fear, sadness, and joy) and 2 sentiments (positive or negative). Mollema et al [134] performed sentiment analysis based on Vasterman and Ruigrok’s classification on the information more objective than judgmental in online newspaper papers. Keim-Malpass et al [44] recorded Topsy, which uses NLP to deliver its sentiment score. Boon-Itt and Skunkan [84] used the National Research Council (NRC) sentiment lexicon for sentiment analysis to examine the expression of Twitter users who followed COVID-19 information. However, 1 (5.3%) study [61] collected 11 sentiment analysis tools currently commonly used on social media data sets and conducted qualitative error analysis on social media posts misclassified by all tools. The results showed that existing sentiment analysis tools perform poorly, and the consistency between tools is also low.

#### Topic Modeling

Of 46 studies, 3 (6.5%) used topic modeling to analyze the contents of social media. Muralidhara and Paul [116] applied the multilingual topic model to 3 types of data (captions, hashtags, and image tag features) and provided different views of each topic. The output topic model was qualitatively interpreted for content analysis. Krawczyk et al [82] used topic modeling to identify COVID-19–related content to quantify the proportion of total coverage the pandemic received in 2020. Cameron et al [85] developed a novel semantic web platform modeled in the manually created drug abuse ontology (DAO) to facilitate the extraction of semantic information from user-generated content.

#### Other NLP Technologies

Of 46 studies, 6 (13.0%) used other NLP technologies to analyze the contents of social media. Odlum and Yoon [121] used N-gram to cluster similar content for topic detection and used an information graph to visualize the clustering to summarize the detected topics. Litchman et al [136] used a proprietary NLP algorithm to extract subjective words and emoticons to determine the level of negativity and positivity. Hussain et al [161] used the sentiment word cloud and N-gram analysis, respectively, near the inflection point of the emotional trend map to determine the topic of discussion and to deeply understand the positive and negative contents of online discourse. Analysis was also conducted throughout the study to identify potential topics and themes. Lao et al [165] used Linguistic Inquiry and Word Count (LIWC) 2015 and the TextStat Python library to extract linguistic features from the average of all posts, thus constructing some machine learning model, such as random forest, gradient boost, and SVM, to evaluate and better understand a person's suicide risk on social media. Jiang et al [110] used a technique to cluster words quantitatively, called the meaning extraction method with principal component analysis (MEM/PCA), and assigned a descriptive topic to each cluster according to the words in the cluster. Hriberšek et al [103] calculated the absolute frequency and relative frequency of single-word and double-word groups in the data set in the form of a word cloud. Furthermore, a Markov chain was generated to show the correlation between 2 words.

### Categories of Research Content

**Figure 3:**
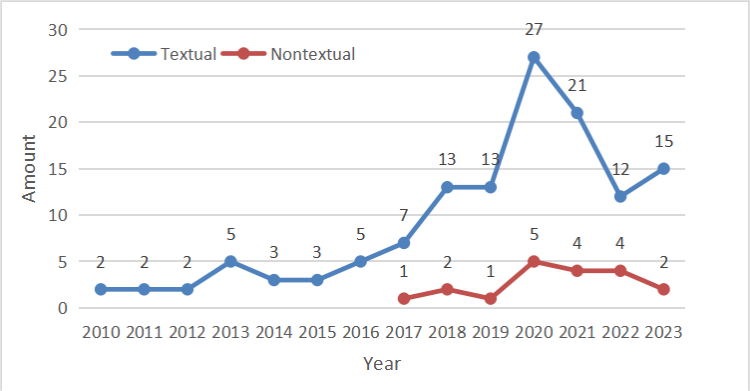
Text content types of included studies.

The main category of research information was online text. Although only about one-seventh (19/134, 14.2%) of the included literature involved nontext-type information research, this quantity was on the rise every year. The trend chart can be seen in Figure 3. In this review, the nontext types of information research were pictures and videos. Among studies focusing on pictures, van der Pijl et al [106] downloaded and saved pictures, then transcribed the text content of each picture into a document, and finally conducted qualitative content analysis. In addition, 4 (21.1%) studies [99,111,152,154] manually extracted image content and encoded it for subsequent content analysis. Zhang et al [81] used image analysis to explore the correlation between the characteristics of pictures posted on WeChat and the risk of postpartum depression. Muralidhara and Paul [116] extracted “tag” features from each image and treated them as an additional type of text, along with captions and hashtags. The polylingual topic model was applied to this type of data and provided corresponding topic views. In studies involving video, the scoring table was the most common analysis method. Researchers corresponded video content and quality to specific rating scales and used statistical methods to calculate and analyze scores. DISCERN criteria [34,69,77,94,124,130,151] and the GQS [69,130,141] were the most commonly used scales. Other qualitative methods were also commonly used, in addition to scoring tables. Abdoli et al [137] transcribed all included videos word for word and analyzed them using qualitative content analysis methods. Fowler et al [73] adopted a retrospective and inductive approach to summarize encoded videos about sex education to identify key themes and subthemes. Kelly-Hedrick et al [107] used a deductive method to encode a video and evaluate its reliability. In addition, Rael et al [131] input a video into a Python script and used the sentiment language vocabulary system to identify a group of words and phrases for manual coding.

### Health Care Area

#### Health Practice

In this review, based on the existing literature [168], we defined health practices as the behaviors or thoughts of online users (mainly patients) on social media regarding a certain disease. Most of the included studies (94/134, 70.1%) aimed to understand the general situation of patients in specific disciplines by identifying intuitive behaviors that appear in relevant medical information, such as sharing experiences and seeking advice. For example, Mehta et al [122] analyzed nursing experiences and concerns by identifying and describing the experiences and exchange of suggestions between patients with Alzheimer disease and their families. Watts et al [127] extracted the 1000 most recent posts on Twitter with relevance to a combined orthodontic and orthognathic surgical treatment and determined the patients’ thoughts, concluding that the 3 main themes identified in relation to orthognathic surgery are preoperative engagement, postoperative difficulties, and posttreatment satisfaction.

#### Health Services

Health services refer to the collection and analysis of emotions, characteristics, or trends related to online medical information from medical professionals to obtain clinical insights or services [169]. Some of the included studies [36,44,45,54,56,​^59^,^74^,^81^,^82^,^86^,^95^,^99^,^136^,^143^,^150^,^161^] used manual or mechanical methods to categorize emotions in posts into positive and negative categories in order to indirectly reflect the views of participants. For example, after investigating the characteristics of pictures posted by mothers after childbirth and exploring the correlation with the risk of postpartum depression, Zhang et al [81] found that women who post selfies after childbirth are more likely to suffer from postpartum depression, which is beneficial for the early identification of and intervention in postpartum depression. Some of the studies [98,146,156,165] improved analysis methods to achieve a higher accuracy in predicting the trends of information on the network and applied them to clinical practice. Other studies [55,60,75,84,85,100,123,147] identified general characteristics and development trends of specific health service content through epidemiological investigation methods. For example, after reviewing the posts related to COVID-19 on Chinese social media platforms, Zhang et al [147] found that the COVID-19 information on Chinese social media is characterized by gradual progress and repeated fluctuations.

#### Health Education

Health education [170] usually affects patients in relation to disease knowledge, symptoms, prevention and treatment guidance, and error information [34,41,67,69,73,77,​^94^,^97^,^107^,^119^,^124^,^130^,^138^,^141^,^151^]. In patient-oriented health education materials published by professionals and institutions, video is the main form and the overall quality is not satisfactory [67,77,94,130,141,151].

## Discussion

### Principal Findings

Our aim was to synthesize methods for analyzing the contents of social media for health care to provide recommendations for future research and practice. A total of 134 studies were included in this scoping review. We found that most of the methods were based on traditional qualitative content analysis. The main category of research information was online text.

Manual content analysis is still the most commonly used method to analyze social media information for health care. As 1 of the most widely used communication research methods, content analysis also plays an important role in the field of health management by classifying information content, analyzing and summarizing the characteristics and interrelationships of various analysis dimensions, and drawing conclusions about the trends or characteristics of research objects [171]. Content analysis has the following advantages: First, after analyzing 64 studies using content analysis, we realized that as a widely used standard method, the category definition and operating rules of content analysis are clear and comprehensive, which requires researchers to follow a predetermined plan step by step (and sometimes calculate and evaluate errors between encoders), and the results are reliable and practical [172]. Second, in the review results, manual content analysis is often used as a control group to compare with the results of computer-aided analysis. It is generally believed that compared to computer-aided analysis, manual analysis has higher accuracy and is superior in handling subtle concepts such as ideology, concepts, value, and meaning [173]. In addition, by reviewing 2 papers [104,142] that used quantitative content analysis, we found that similar to computer-aided analysis, content analysis can also identify the essential features of content to which statistics can be easily applied through quantitative analysis of information, thereby achieving a more accurate understanding of information [174].

However, content analysis also has several shortcomings. First, ordinary manual content analysis is mainly suitable for small samples, which range from 32 to 63,770, with an average of 4668. Obviously, with large samples, it is tedious and time-consuming to classify and code the content compared to computer-aided methods. At the same time, there is still doubt about whether application content analysis can fully meet the massive medical information in the era of big data. Second, we reviewed and found that content analysis is mainly applied to network text analysis, but it has not been widely used in analyzing nontextual network information, such as pictures and videos. Given the diverse sources of online health care information (eg, text, voice, images, videos, physiological signals), although the textual form is still the mainstream online information type, other types of information have also been proven to be unique [81]. Focusing solely on analyzing textual content clearly does not meet future development trends. Third, most qualitative research papers in this field have focused on analyzing and summarizing data content, emphasizing common content, and not valuing the digital foundation. Content analysis mainly emphasizes the expression of words and language, emphasizing a deep understanding and explanation of the essence and connotation of phenomena [171]. Compared with nonmanual methods, the demographic characteristics of a large amount of information are not easy to quantify. Finally, content analysis is a description of the content of communication, which can prove the correlation between variables but rarely reveals the causal relationship. In addition, we found that content analysis and thematic analysis sometimes have conceptual confusion in the literature we included. Since the difference between the 2 in information research and the impact on the results are not significant [175], we did not strictly distinguish and compare the 2 methods.

In the past 5 years, there has been an increase in research on the existing information about health care on social media compared to the past. The world is slowly becoming a technology-driven place [3]. Therefore, it is necessary to adopt trendy internet analysis technology. Gradually enriching research is beneficial for researchers to deepen their understanding and analysis of health care information, design better intervention methods to collect and respond to patients based on the role of information, and provide a foundation for faster implementation of intelligent health care in the future, which is conducive to promoting the progress and development of big data health care [176]. However, our research also found that there is a lack of research on nontextual information on social media for health care. In view of the diversified process of social media information sources, only focusing on content analysis in the form of text cannot meet our needs. Bazzaz et al [31] classified a content-oriented thematic learning method, focusing on the communication content of social networks, including text mining, video content analysis, and image analysis. The aim was to discover potential similarities and hidden associations and convert them into structured data for further analysis.

### Limitations

Some limitations were identified in this scoping review. Due to time and translation reasons, we only selected papers published in English, which may lead to some bias. Moreover, we did not conduct a formal risk assessment of bias; therefore, the selection of all related papers cannot be guaranteed. In addition, the research included was divided into 2 categories according to whether the analysis method was manual analysis, which may lead to some prejudice.

### Conclusion

The main contribution of this review was to collect and summarize the methods for analyzing medical and health information that have been applied to social media and divide them into groups according to the study methods, strategies, and different health care areas. We identified 134 papers published between 2010 and May 2023 that were relevant to our review, which can be conducive to understanding the status and trends in method usage. In conclusion, we noticed that most of the included studies used traditional manual content analysis methods. At the same time, some studies also relied on computer-aided technical analysis to understand health information through artificial intelligence methods. Based on the studies we included, although there are relatively few studies on using machine learning to analyze the contents of social media, this number has increased gradually in recent years, which is consistent with the development and progress of the internet era. This review can guide us in designing studies to investigate the existing health management information on social media and analyze the research content from qualitative and quantitative perspectives.

With the progress of computers, mobile phones, smart watches, and other smart devices, social media information sources will become more diversified. Future research can combine new sources, such as pictures, videos, and physiological signals, with online social networking to adapt to the development trend of the internet. With the advent of the internet era, the medical industry has been inevitably linked with social media. However, at present, the number of information management talents in the field of medical care is not satisfactory. We hope that the findings of this review will help researchers investigate the health information on the internet so as to overcome the challenges of social big data.

## Appendix

Multimedia Appendix 1 Related reviews for social media.

Multimedia Appendix 2 Search strategies.

Multimedia Appendix 3 Inclusion and exclusion criteria.

Multimedia Appendix 4 Descriptions of the included literature.

## Abbreviations

GQS: Global Quality Scale
LDA: latent Dirichlet allocation
NLP: natural language processing
SVM: support vector machine

## Data Availability

All data generated or analyzed during this study are included in this paper and its supplementary material.
