# Supplementary material for: Methods for Analyzing the Contents of Social Media for Health Care: Scoping Review
Source: J Med Internet Res. 2023 Jun 26;25:e43349. doi: 10.2196/43349 (PMC10337469; doi:10.2196/43349)
Supplement: Multimedia Appendix 1 [file jmir_v25i1e43349_app1.docx]

Table S1. Related reviews for Social Media

| Work | Area of Focus | Contribution(s) |
| --- | --- | --- |
| Farsi [15] | The application in health care providers or patients | Highlighs on SM use worldwide and discusses how it has been used as an essential tool in the health care industry from the perspective of HCPs. |
| Farsi [16] |  | Discusses how social media has been used in the health care industry from the perspective of patients. |
| Frey [17] |  | Examines 1) How do parents use social media to find health information for their children? 2) What motivates parents to engage with social media to seek health information for their children? 3) How do parents seek to understand and evaluate the health information? |
| Grajales [18] |  | Synthesizes the impact and illustrate, explain, and provides contextual knowledge of the applications and potential implementations of social media in medicine and health care. |
| Alonzo [19] | The relationship with mental health | Provides a comprehensive assessment on the relationship between active social media use, sleep quality, and common mental health outcomesamong youth. |
| Karim [20] |  | Analyzes the impact of social media activities on two mental health outcomes. |
| Rocha [21] |  | Observes that infodemic knowledge can cause psychological disorders and panic, fear, depression, and fatigue. |
| Teague [22] |  | Examines mental health during disasters or crises by using social media data. |
| Alvarez-Jimenez [23] | The role in online health promotion | Systematically compiles and analyzes the evidence on the acceptability, feasibility, safety and benefits of online and mobile-based interventions for psychosis. |
| Chen [24] |  | Summarizes 10 social media uses for various health purposes by health institutions, health researchers and practitioners, and the public. |
| Sivaratnam [25] |  | Identify social media strategies that enhance participation in priority-setting research, collate metrics assessing the effectiveness of social media campaigns, and summarizes the benefits and limitations of social media-based research approaches, as well as recommendations for prospective campaigns. |
| Afful-Dadzie [26] | The feature of social media health information | (1) Health topics, users and social media platforms that have raised health information quality concerns are reviewed. (2) The review also looked at the suitability of existing criteria and instruments used in evaluating SMHI and identified gaps for future research. |
| Bour [27] |  | Provides an evidence map of the different uses of social media for health research purposes, their fields of application, and their analysis methods. |
| Gunasekeran [28] |  | Highlights a brief history of social media in health care and report its potential negative and positive public health impacts |
| Suarez-Lledo [29] |  | Identify the main health misinformation topics and their prevalence on different social media platforms, focusing on methodological quality and the diverse solutions |
| Alshaikh [30] | Big data analysis methods | Explores the use of SNS as a mode of collecting data for health research. |
| Bazzaz [31] |  | Reviewes the big data analysis methods in social networks and divided them into two categories: content-oriented methods and network-oriented methods. |
| Etemadi [32] |  | Identify, taxonomically classify, and compare current HRS researches in a systematic way. |
| Ismail [33] |  | (1) provides an in-depth analysis of the BcC integration for the healthcare system. (2) reviews the development platforms and services and highlight the research challenges for the integrated BcC architecture, possible solutions, and future research directions. |
