# Supplementary material for: Methods for Analyzing the Contents of Social Media for Health Care: Scoping Review
Source: J Med Internet Res. 2023 Jun 26;25:e43349. doi: 10.2196/43349 (PMC10337469; doi:10.2196/43349)
Supplement: Multimedia Appendix 2 [file jmir_v25i1e43349_app2.docx]

**Search strategy for PubMed**

#1 social media [MeSH Terms] 15434

#2 “social network site*” [Title/Abstract] 351

#3 “social media” [Title/Abstract] 28147

#4 facebook [Title/Abstract] 6403

#5 twitter [Title/Abstract] 6433

#6 linkedin [Title/Abstract] 342

#7 instagram [Title/Abstract] 1776

#8 weibo [Title/Abstract] 452

#9 whatsapp [Title/Abstract] 1605

#10 telegram [Title/Abstract] 192

#11 wechat [Title/Abstract] 1075

#12 “online community” [Title/Abstract] 871

#13 #1 OR #2 OR #3 OR #4 OR #5 OR #6 OR #7 OR #8 OR #9 OR #10 OR #11 OR #12 (39374)

#14 [Delivery of Health Care](https://www.ncbi.nlm.nih.gov/mesh/68003695) [MeSH Terms] 1223660

#15 “health care” [Title/Abstract] 454289

#16 nurs* [Title/Abstract] 529425

#17 “health management” [Title/Abstract] 7997

#18 #14 OR #15 OR #16 OR #17 (1877532)

#19 #13 AND #18 (8439)

#20 random* [Title/Abstract] 1417310

#21 quantitative* [Title/Abstract] 856413

#22 qualitative [Title/Abstract] 319751

#23 interview [Title/Abstract] 167609

#24 clinical trial* [Title/Abstract] 485894

#25 surve* [Title/Abstract] 1045665

#26 “descriptive study” [Title/Abstract] 34873

#27 “cross-sectional stud*” [Title/Abstract] 254368

#28 "content analysis" [Title/Abstract] 42227

#29 phenomenology [Title/Abstract] 11512

#30 "grounded theory" [Title/Abstract] 14874

#31 #20 OR #21 OR #22 OR #23 OR #24 OR #25 OR #26 OR #27 OR #28 OR #29 OR #30 (3967982)

#32 #19 AND #31 (5821)

**Search strategy for Web of Science**

#1 TS=social media

#2 TI=“social network site*”

#3 TI=“social media”

#4 TI=facebook

#5 TI=twitter

#6 TI=linkedin

#7 TI=instagram

#8 TI=weibo

#9 TI=whatsapp

#10 TI=telegram

#11 TI=wechat

#12 TI=“online community”

#13 #1 OR #2 OR #3 OR #4 OR #5 OR #6 OR #7 OR #8 OR #9 OR #10 OR #11 OR #12 (34052)

#14 TS=[Delivery of Health Care](https://www.ncbi.nlm.nih.gov/mesh/68003695)

#15 TS=“health care”

#16 TS=nurs*

#17 TS=“health management”

#18 #14 OR #15 OR #16 OR #17 (2003091)

#19 #13 AND #18 (9233)

#20 TS=random*

#21 TS=quantitative*

#22 TS=qualitative

#23 TS=interview

#24 TS=clinical trial*

#25 TS=surve*

#26 TS=“descriptive study”

#27 TS=“cross-sectional stud*”

#28 TS="content analysis"

#29 TS=phenomenology

#30 TS="grounded theory"

#31 #20 OR #21 OR #22 OR #23 OR #24 OR #25 OR #26 OR #27 OR #28 OR #29 OR #30 (7602212)

#32 #18 AND #30 (3851)

**Search strategy for Cochrane Library**

#1 MeSH descriptor: [social media] explode all trees 563

#2 “social network site*”:ti,ab,kw 3

#3 “social media”:ti,ab,kw 2746

#4 facebook:ti,ab,kw 947

#5 twitter:ti,ab,kw 227

#6 linkedin:ti,ab,kw 16

#7 instagram:ti,ab,kw 204

#8 weibo:ti,ab,kw 11

#9 whatsapp:ti,ab,kw 672

#10 telegram:ti,ab,kw 110

#11 wechat:ti,ab,kw 521

#12 “online community”:ti,ab,kw 114

#13 #1 OR #2 OR #3 OR #4 OR #5 OR #6 OR #7 OR #8 OR #9 OR #10 OR #11 OR #12 4269

#14 MeSH descriptor: [[Delivery of Health Care](https://www.ncbi.nlm.nih.gov/mesh/68003695)] explode all trees 61019

#15 “health care”:ti,ab,kw 74726

#16 nurs*:ti,ab,kw 53345

#17 “health management”:ti,ab,kw 592

#18 #14 OR #15 OR #16 OR #17 158181

#19 #13 AND#18 994

#20 random*:ti,ab,kw 1234274

#21 quantitative*:ti,ab,kw 32139

#22 qualitative:ti,ab,kw 20605

#23 interview:ti,ab,kw 27726

#24 clinical trial*:ti,ab,kw 881042

#25 surve*:ti,ab,kw 85523

#26 “descriptive study”:ti,ab,kw 811

#27 “cross-sectional stud*”:ti,ab,kw 0

#28 "content analysis":ti,ab,kw 1710

#29 phenomenology:ti,ab,kw 203

#30 "grounded theory":ti,ab,kw 431

#31 #20 OR #21 OR #22 OR #23 OR #24 OR #25 OR #26 OR #27 OR #28 OR #29 OR #30 1391238

#32 #19 AND #31 875

**Search strategy for Embase**

#1 ’social media’:ab,ti 33039

#2 ’social network site*’:ab,ti 339

#3 facebook:ab,ti 9037

#4 twitter:ab,ti 7647

#5 linkedin:ab,ti 598

#6 instagram:ab,ti 2342

#7 weibo:ab,ti 364

#8 whatsapp:ab,ti 2363

#9 telegram:ab,ti 239

#10 wechat:ab,ti 1142

#11 ’online community’:ab,ti 1139

#12 #1 OR #2 OR #3 OR #4 OR #5 OR #6 OR #7 OR #8 OR #9 OR #10 OR #11 43890

#13 '[delivery of health care](https://www.ncbi.nlm.nih.gov/mesh/68003695)':ab,ti 2355

#14 'health care':ab,ti 522260

#15 nurs*:ab,ti 625053

#16 'health management':ab,ti 9236

#17 #13 OR #14 OR #15 OR #16 1091782

#18 #12 AND #17 5179

#19 random*:ab,ti 1920632

#20 quantitative*:ab,ti 1055442

#21 qualitative:ab,ti 383813

#22 interview:ab,ti 218639

#23 surve*:ab,ti 1348495

#24 ’clinical trial*’:ab,ti 679264

#25 ’descriptive study’:ab,ti 53647

#26 ’cross-sectional stud*’:ab,ti 328480

#27 ’content analysis’:ab,ti 47124

#28 ’grounded theory’:ab,ti 18261

#29 phenomenology:ab,ti 11997

#30 #19 OR #20 OR #21 OR #22 OR #23 OR #24 OR #25 OR #26 OR #27 OR #28 OR #29 5191323

#31 #18 AND #30 2989

**Search strategy for CINAHL**

#1 TI social media 30502

#2 TI social network site 1499

#3 TI facebook 1465

#4 TI twitter 1251

#5 TI linkedin 47

#6 TI instagram 381

#7 TI weibo 24

#8 TI whatsapp 131

#9 TI telegram 12

#10 TI wechat 154

#11 #1 OR #2 OR #3 OR #4 OR #5 OR #6 OR #7 OR #8 OR #9 OR #10 32558

#12 AB [delivery of health care](https://www.ncbi.nlm.nih.gov/mesh/68003695) 23835

#13 AB health care 298195

#14 AB nurs* 289860

#15 AB health management 79722

#16 #12 OR #13 OR #14 OR #15 568446

#17 #11 AND #16 5468

#18 AB random* 323709

#19 AB quantitative* 90578

#20 AB qualitative 123014

#21 AB interview 181317

#22 AB surve* 287632

#23 AB clinical trial* 146589

#24 AB descriptive study 75193

#25 AB cross-sectional stud* 140975

#26 AB content analysis 49182

#27 AB grounded theory 11505

#28 AB phenomenology 3329

#29 #18 OR #19 OR #20 OR #21 OR #22 OR #23 OR #24 OR #25 OR #26 OR #27 OR #28 1026664

#30 #17 AND #29 2625
