# Supplementary material for: Methods for Analyzing the Contents of Social Media for Health Care: Scoping Review
Source: J Med Internet Res. 2023 Jun 26;25:e43349. doi: 10.2196/43349 (PMC10337469; doi:10.2196/43349)
Supplement: Multimedia Appendix 3 [file jmir_v25i1e43349_app3.docx]

Table 1. Inclusion and exclusion criteria

| Criterion | Inclusion | Exclusion |
| --- | --- | --- |
| Types of studies | original studies including experimental and observational methods. | Letters, comments, conference abstracts, editorials, doctoral thesis, or any type of review. |
| Language | English. | All other languages. |
| Analysis object | Existing information on the social media. | Non Information Content. |
